# Resequencing core accessions of a pedigree identifies derivation of genomic segments and key agronomic trait loci during cotton improvement

**DOI:** 10.1111/pbi.13013

**Published:** 2018-10-18

**Authors:** Xiongfeng Ma, Zhenyu Wang, Wei Li, Yuzhou Zhang, Xiaojian Zhou, Yangai Liu, Zhongying Ren, Xiaoyu Pei, Kehai Zhou, Wensheng Zhang, Kunlun He, Fei Zhang, Junfang Liu, Wenyu Ma, Guanghui Xiao, Daigang Yang

**Affiliations:** ^1^ State Key Laboratory of Cotton Biology Institute of Cotton Research Chinese Academy of Agricultural Sciences Anyang China; ^2^ Key Laboratory of the Ministry of Education for Medicinal Plant Resources and Natural Pharmaceutical Chemistry National Engineering Laboratory for Resource Development of Endangered Crude Drugs in the Northwest of China College of Life Sciences Shaanxi Normal University Xi'an China; ^3^ Present address: College of Life Sciences Shaanxi Normal University Xi'an China; ^4^ Present address: Institute of Cotton Research Chinese Academy of Agricultural Sciences Anyang China

**Keywords:** modern cotton improvement, cotton pedigree, identity by descent (IBD), lint percentage, resequencing

## Abstract

Upland cotton (*Gossypium hirsutum*) is the world's largest source of natural fibre and dominates the global textile industry. Hybrid cotton varieties exhibit strong heterosis that confers high fibre yields, yet the genome‐wide effects of artificial selection that have influenced Upland cotton during its breeding history are poorly understood. Here, we resequenced Upland cotton genomes and constructed a variation map of an intact breeding pedigree comprising seven elite and 19 backbone parents. Compared to wild accessions, the 26 pedigree accessions underwent strong artificial selection during domestication that has resulted in reduced genetic diversity but stronger linkage disequilibrium and higher extents of selective sweeps. In contrast to the backbone parents, the elite parents have acquired significantly improved agronomic traits, with an especially pronounced increase in the lint percentage. Notably, identify by descent (IBD) tracking revealed that the elite parents inherited abundant beneficial trait segments and loci from the backbone parents and our combined analyses led to the identification of a core genomic segment which was inherited in the elite lines from the parents Zhong 7263 and Ejing 1 and that was strongly associated with lint percentage. Additionally, SNP correlation analysis of this core segment showed that a non‐synonymous SNP (A‐to‐G) site in a gene encoding the cell wall‐associated receptor‐like kinase 3 (GhWAKL3) protein was highly correlated with increased lint percentage. Our results substantially increase the valuable genomics resources available for future genetic and functional genomics studies of cotton and reveal insights that will facilitate yield increases in the molecular breeding of cotton.

## Introduction

The *Gossypium* genus, which originated from a paleo‐hexaploid eudicot, has evolved into various species during its evolutionary history, including approximately 45 diploid and five tetraploid species (Brubaker *et al*., 1999; Fryxell, 1992). Further, the genome has also diversified into eight diploid groups, including A‐G and K (Paterson *et al*., 2012; Wendel *et al*., 2009). In particular, Upland cotton (*Gossypium hirsutum*), the largest natural source of industrial fibre, plays a predominant role in the supply of cotton fibre, which is estimated to comprise more than 90% of the fibre produced by cultivated cotton worldwide (Campbell *et al*., 2010; Chen *et al*., 2007). Allotetraploidal cotton is postulated to be around 1.5 million years old, and has been continuously domesticated over the past 4000–5000 years by humans (Wang *et al*., 2017; Wendel, 1989). It was introduced into China in the mid‐19th century for the first time, and since the 1919 many cotton elite germplasms have been brought into China with the aim to improve both fibre yield and quality using traditional breeding. Several outstanding representative species from The United States have been introduced into China, including Lone Star, Deltapine 15, and Stoneville 2B, or breeding efforts aimed at developing new Upland cotton varieties (Fang *et al*., 2017b; Liu *et al*., 2003). For example, using these germplasm, many Upland cotton accessions with excellent quality and yield, including Xuzhou 209, Guannong 1, CCRI 2, Simian 2, 86‐1, Ejing 1 and CCRI 12, have been successfully bred (Fang *et al*., 2017b; Liu *et al*., 2003). These accessions, together with newer introduced varieties, have provided the necessary genetic resources for modern breeding programmes to replace original diploid cotton varieties grown in China. The critical value of these genetic feedstocks has gained world‐wild recognition for the development of new lines and hybrids. In both maize and rice, it has been shown that using high‐quality backbone parents significantly improves breeding efficiency (Shinada *et al*., 2014; Smith, 2007; Smith *et al*., 2004; Wu *et al*., 2014; Zhou *et al*., 2016a). The benefits of using these backbone parents is that they not only represent the most excellent haplotype blocks in breeding, but also have abandoned several harmful genotypes associated with negative traits during the improvement process (Voss‐Fels *et al*., 2017). Utilization of plants with a complete pedigree relationship not only facilitates interpretation of the genetic characteristics of the backbone parents, but also provides a traceable and clear genetic route for sources of genomic components (Zheng *et al*., 2017; Zhou *et al*., 2016a). Using the appropriate molecular markers, we can now comprehensively analyse the genetic makeup and track the derivation through detection of traceable identity by descent (IBD) fragments, which are genomic segments inherited from their ancestral parents (Fang *et al*., 2017b; Wu *et al*., 2016). In addition, pedigree‐based selective sweep analysis is also able to reveal selective signatures that have arisen during modern crop improvement and identify genomic regions related to artificial selection (Chen *et al*., 2017; Doebley *et al*., 2006).

In recent years, genome‐wide resequencing has become an extensive and effective strategy to identify genetic variation, and has been performed on various species to study their evolution, diversification, domestication and environmental adaptability (Chen *et al*., 2016; Chung *et al*., 2014; Kim *et al*., 2010; Li *et al*., 2016a; Lin *et al*., 2015; Yang *et al*., 2016; Zhou *et al*., 2016b). In plants, this strategy has also been effectively used to identify multiple types of variations, such as SNPs (single‐nucleotide polymorphisms), Indels (insertions and deletions), SVs (structural variations) and CNVs (copy number variations). These variations can be subsequently used for quantitative genetics studies, including the generation of high‐density genetic linkage maps and quantitative trait loci (QTL) and genome‐wide association (GWAS) analyses (Li and Erpelding, 2016; Walford *et al*., 2016; Zhang *et al*., 2016; Zhou *et al*., 2016c). Moreover, genome‐wide resequencing also provides an opportunity for genetic analysis of the backbone parents and for studying the rapid recombination of genomic segments in a conformed relationship pedigree during crop breeding (Wu *et al*., 2016). For example, according to combined pedigree and SNP information, the shift of genomic structure and history of genetic architecture during breeding improvement of Huanghuazhan, a famous rice backbone parent, were revealed, and the major effectual genomic regions were pinpointed to the derivation of traceable genomic blocks (Chen *et al*., 2017; Zhou *et al*., 2016a). Based on pedigree resequencing, Lai *et al*. analysed the genetic variation patterns of six elite maize inbred lines, resulting in the mapping the genome‐wide distribution of sequence diversity levels, zero diversity genes and ultimately uncovered 101 low‐sequence diversity genome segments (Lai *et al*., 2010).

Recently, great progress has been made concerning the origin, domestication and evolution of cotton (Brubaker *et al*., 1999; Paterson *et al*., 2012; Tang *et al*., 2015; Wendel *et al*., 2009). Specifically, two sets of genomic sequences of *Gossypium hirsutum* TM‐1 (AADD) have been published, which provided insights into how both genome evolution and domestication contribute to cotton fibre development (Li *et al*., 2015, 2016b; Zhang *et al*., 2015). Fang *et al*. studied the genetic contribution of American cotton lines DPL15, STV2B and Uganda Mian on seven widely grown cultivars in China by detecting IBDs (Fang *et al*., 2017b), and showed that the genetic contributions from the seven cultivars derived from DPL15, STV2B and Uganda Mian were approximately 14.19%, 10.45% and 4.19%, respectively. These data further supported the important role of American cotton in breeding modern cultivars in China. However, little is known about the genome‐wide genetic basis of Upland cotton breeding improvement programmes using artificial selection.

Ekangmian 9 played a major role in our breeding process. Eleven high‐quality cotton cultivars widely grown in China, including CCRI 53, CCRI 55, CCRI 62, CCRI 63, CCRI 65, CCRI 66, CCRI 71, CCRI 87, Ezamain 4, Ezamian 15 and Ezamian 24 were bred using Ekangmian 9 as a parent. Among them, CCRI‐63, CCRI‐66 and Ezamian 24 have been authorized for commercial production by the National Crop Variety Identification Committee, while other varieties have also been approved for growing in several provinces. During this breeding process, several historically famous accessions were used to generate this pedigree, including Lone Star, Deltapine 15, Stoneville 2B, Delfos and Foster, which have been globally used for Upland cotton breeding throughout the past century. The overall qualities of the cultivars generated from the typical breeding process are excellent; however, how genetic flow and key trait regions among this pedigree contribute to these qualities remained obscure. In this study, 26 elite Upland cotton accessions from a complete pedigree of Ekangmian 9 were deeply resequenced to reveal the genetic patterns that arose during modern cotton improvement efforts. Using these resequencing results, 2 121 046 high‐quality SNPs were identified, with an average of 90.7 SNPs per 100 kb. 647 conserved segments without diversity and 511 selective sweeps were identified, revealing selected genomic segments within the pedigree. 203.14, 327.40 and 565.71 Mb core segments, which can be clearly traced to the original parent, were obtained in Ejing 1, Zhong 7263 and Ekangmian 9, revealing the genomic flow in the pedigree, respectively. According to the common IBD fragments in seven elite parents inherited from Ekangmain 9, a key trait locus associated with lint percentage was identified, which was supported by both expression profiles and candidate association analyses.

## Results

### Pedigree and trait improvement

**Figure 1:**
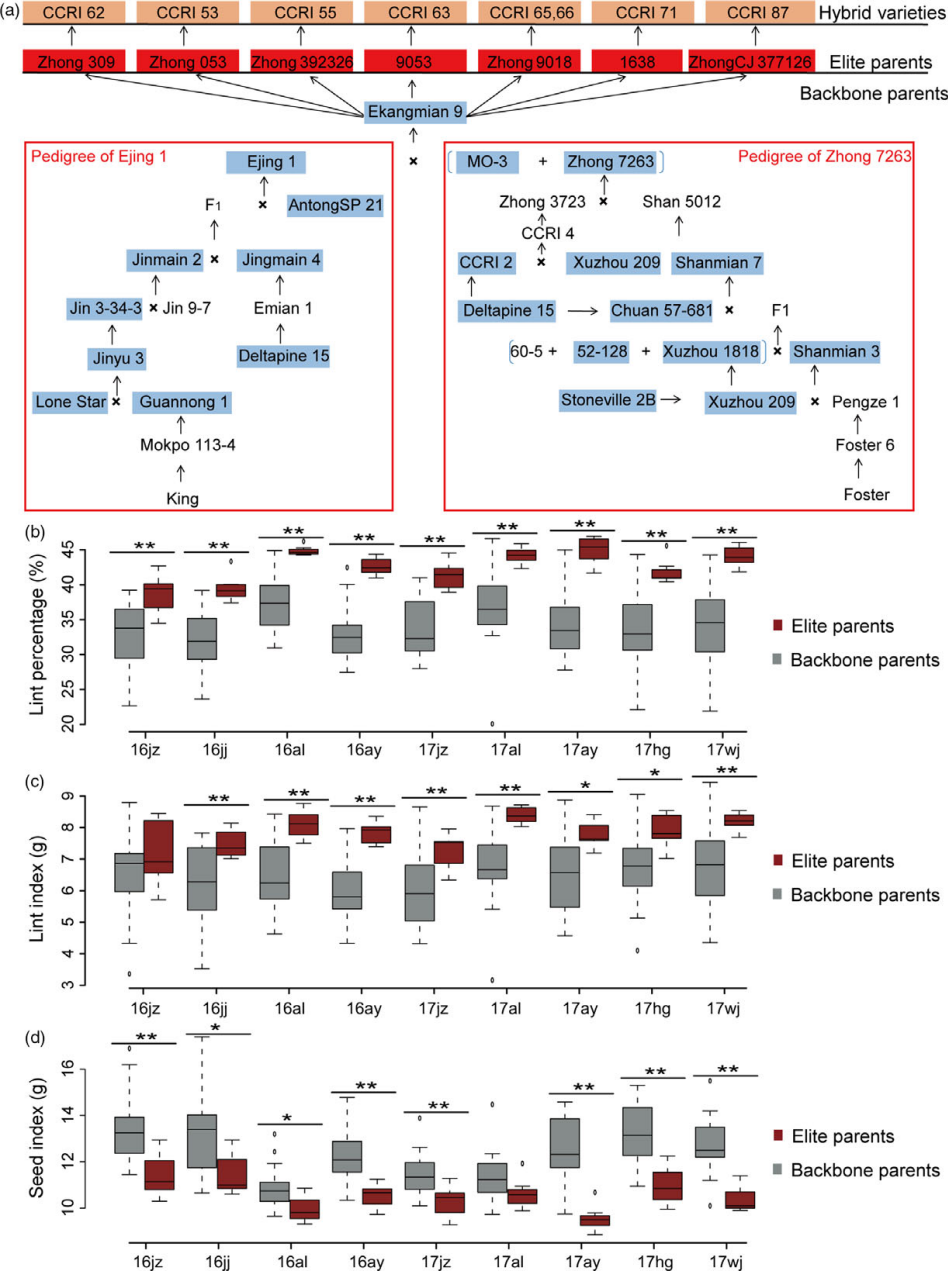
Pedigree of the elite cotton cultivar Ekangmian 9, and statistical analysis of lint percentage, lint index and seed index of the pedigrees in four different locations. (a) Seven elite strains (red) were derived from Ekangmian 9. These elite strains were used as elite parents to breed several hybrid cotton varieties (bright orange). Seven elite parents and 19 backbone parents (blue) were selected for resequencing in this study. Analysis of (b) lint percentage (%), (c) lint index (grams) and (d) seed index (grams) of backbone and elite parents of the cotton harvested from nine environments in China. Dark red and grey indicate elite and backbone parents, respectively. Centre lines indicate medians; box limits represent upper and lower quartiles; and whiskers delineate 1.5× the interquartile range (*P < 0.05, **P < 0.01, two‐sided *t*‐test).

Eight widely used hybrid cotton cultivars were successfully bred by our group using seven elite strains derived from Ekangmian 9 as parents. To clearly show the breeding process of these excellent cotton cultivars, a schematic pedigree was drawn (Fig. 1a). Ejing1, Zhong 7263, MO‐3, and the parents of Ekangmian 9 were successfully bred using several historically famous cultivars. These cultivars include Lone Star and Deltapine 15, which are the parents of Ejing 1, and Stoneville 2B, Delfos and Foster, the parents of Zhong 7263.

To investigate yield and quality traits of different accessions in the pedigree, 19 backbone cultivars and seven elite parents were planted in four different locations in 2016, including Jingzhou, Jiujiang, Anyang and Alaer, and were planted in five different locations in 2017,including Jingzhou, Wangjing, Anyang, Huanggang and Alaer. These locations include the three major areas of cotton cultivation in China. Subsequently, 13 agronomic traits were measured, including growth period, plant height, fruit branch number, boll number, boll weight, lint percentage, seed index, lint index, fibre upper‐half mean length, fibre uniformity, fibre strength, micronaire and fibre elongation (Table S1). In these traits, compared with the backbone parents, the lint percentage and lint index of elite parents showed a trend of improvement and the seed index of elite parents showed a trend of decline (Figure S1a,b and c). Among them, lint percentage of elite parents was extremely significantly higher than that in backbone cultivars in all nine investigated environments (Fig. 1b and Table S2). Lint index of elite parents were significantly higher than that in backbone cultivars in eight investigated environments (Fig. 1c and Table S2).However, seed index of elite parents was significantly lower than that of backbone cultivars in eight investigated environments (Fig. 1d and Table S2). Meanwhile, there was no extremely significant change in fibre quality traits between elite and backbone parents in these locations (Table S2). These results suggest that lint percentage has been selected as a major target in the breeding process of elite parents, and that increased lint index combined with decreased seed index are the primary contributors for increased lint percentage (The seed index was the weight of 100 seeds and the lint index was the lint obtained from 100 seeds. Lint percentage = lint weight/seed cotton weight) (Munir *et al*., 2016).

### Re‐sequencing‐based SNP calling and selective sweeps analysis

In order to explore genetic variations and analyse genetic patterns that arose during the breeding process, 26 representative varieties in an intact pedigree, including 19 backbone cultivars and seven elite parents used for the yield and quality trait analyses above, were selected for whole genome resequencing. A total of 2.27 Tb of clean data was obtained with high‐quality values (Q20 was 94.93% and Q30 was 88.91% on average) and an average depth of 34.65× (Table S3). An average of 99.37% of the reads was mapped to the genome, ranging from 98.92% to 99.71%. 97.00% of the reference genome was covered by reads, with 93.25% covered with at least 4× depth on average.

SNP detection was performed for each sample based on this high‐quality resequencing data. The number of SNPs detected per sample varied from 1 699 580 to 2 685 167, with an average of 2 121 628 (Table S4), indicating that there is an abundant diversity of SNPs in these samples. Furthermore, the Stoneville 2B (SRR5512449) genome was used for genotyping these varieties. As a result, 2 121 046 high‐quality SNPs were identified. Most of them (1 861 221) were located in intergenic regions. Further, 43 842 SNPs were located in the upstream regions of genes, 40 602 in the downstream regions of genes, 116 670 in intronic regions and 55 977 in exonic regions (Figure S2a). The ratio of non‐synonymous‐to‐synonymous (dN/dS) SNPs was used to estimate the evolutionary direction by mutations (Kryazhimskiy and Plotkin, 2008). The dN/dS ratio was 1.58 in the pedigree (Figure S2b), which is higher than that observed in other cultivars (dN/dS 1.46) (Wang *et al*., 2017). This result suggested that more non‐synonymous mutations were beneficial mutations, and therefore retained during cotton cultivation. We further identified several, large‐effect SNPs that resulted in the disruption of open reading frames (ORF) through the insertion or deletion of a stop codon (stop‐gained SNP and stop‐lost SNP). As a result, 851 large‐effect SNPs were found in 785 genes involved in 83 KEGG pathways (Table S5 and S6), of which 704 were stop‐gained SNPs distributed in 638 genes and 147 were stop‐lost SNPs located in 147 genes (Figure S2c and Table S5). In addition, these genes were found to be associated with 1563 GO terms, including biological metabolism, composed cellular components and biological processes (Table S7).

**Figure 2:**
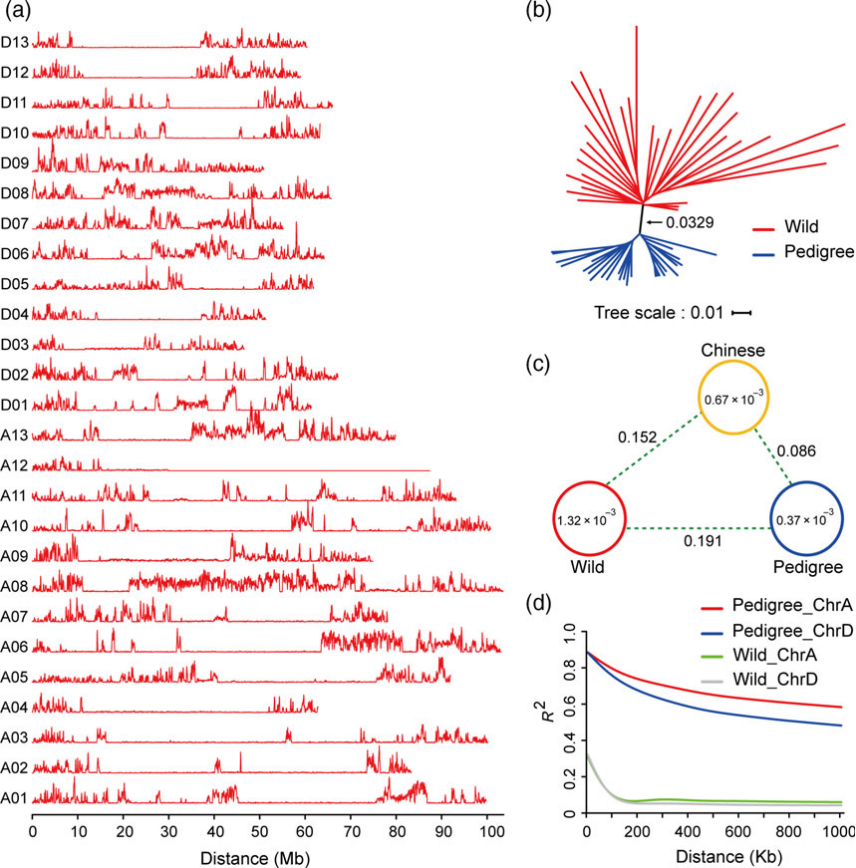
SNP distribution and population diversity between wild and pedigree cotton accessions. (a) SNP density in 27 allotetraploid cotton genomes. The 26 allotetraploid cotton chromosomes, A1–D13, are indicated on the vertical axis. Horizontal curves indicate the density of SNP loci, with the minimum set to 0. The axis of abscissa designates chromosome size in the curves. (b) Phylogenetic analysis of wild type and pedigree groups. The neighbour‐joining tree was constructed using whole genome SNPs from the wild (31 accessions) and pedigree groups (27 accessions or strains). (c) Nucleotide diversity (π) and population divergence (Fst) among the three groups. The value in each circle and line represent nucleotide diversity and population divergence, respectively. The measure of nucleotide diversity for both wild and Chinese groups, and the value population divergence between the two groups were cited from recently published results (Wang *et al*., 2017). (d) Decay of linkage disequilibrium (LD) in the sub‐genome of each group.

SNP density analysis was performed to clearly investigate whole genome SNP distribution across these accessions (Fig. 2a). These results show that SNP density ranged from 0 to 0.01303, with an average of 0.000907. (Table S8). 647 segments without diversity were identified, with the longest one approaching 57 500 Kb, while the shortest was only 100 Kb (Table S9). The longest segment was located in chromosome A12 and comprised up to 65.73% of the entire chromosome. In total, the size of these segments added together was 135.48 Mb, including 95.56 Mb in the A sub‐genome and 39.92 Mb in the D sub‐genome. The much more segments were found in the A sub‐genome might be due to the much larger size of the A sub‐genome, which is nearly 1.5 times size of the D sub‐genome. These segments were found to possess 3137 genes, including 2241 genes in the A sub‐genome and 896 genes in the D sub‐genome (Table S9), which were predicted to be involved in 114 KEGG pathways (Table S10) and associated 2714 GO terms (Table S11). These results suggest that there were a large number of conserved segments in the pedigree, and the A sub‐genome tends to be much more conserved than the D sub‐genome.

To explore the phylogenetic relationships and domestication process within these accessions, 26 accessions in our pedigree together with 31 wild cotton cultivars were used to build a phylogenetic tree from the genome‐wide SNP data. Two distinct taxa were observed between wild cotton and the accessions in our pedigree (Fig. 2b), indicating that a divergence occurred between wild cotton and the pedigrees during domestication in China. Genetic diversity and linkage disequilibrium (LD) have been widely used to analyse genomic polymorphism (Fang *et al*., 2017a; Wang *et al*., 2017). In our pedigrees, nucleotide diversity (π) was 0.37 × 10^−3^, which was significantly lower than wild cotton (1.32 × 10^−3^) (Wang *et al*., 2017), and was even lower than that in the Chinese cultivar group (0.67 × 10^−3^) (Wang *et al*., 2017) (Fig. 2c). Meanwhile, measurements of population fixation statistics (Fst) among these groups show that population divergence between the pedigree group and the wild group was greater than the population divergence between the Chinese group and the wild group, and population divergence between the Chinese group and the pedigree group has also arisen (Fig. 2c). Moreover, linkage disequilibrium in the pedigrees was significantly increased relative to wild cotton (Fig. 2d). These results suggest that the genome of the accessions in our pedigree underwent higher purifying selection pressure than the wild group, which led to the loss of a large amount of genetic diversity in the sub‐genomes during cotton domestication.

The significant genetic differences observed between wild cotton and our pedigree group prompted us to analyse selective sweeps during the improvement process. 517 selective sweeps were identified that were 100–660 Kb in length, occupying 83.98 Mb of the whole genome (42.8 Mb in the A sub‐genome, 41.18 Mb in the D sub‐genome) (Figure S3 and Table S12). 1885 protein‐coding genes, including 663 in the A sub‐genome and 1222 in the D sub‐genome, were located in 416 selective sweeps (Table S12). The difference of genes in selective sweeps may be due to asymmetric domestication selection of A sub‐genome and D sub‐genome (Wang *et al*., 2017). To determine the genetic basis of cotton domestication, we overlapped the selective sweeps with the QTL hotpots. These sweeps were found to be highly overlapped with 69 QTL loci containing many QTLs related to fibre quality (Table S13). These genes were predicted to be involved in 108 KEGG pathways (Table S14) and associated with 2195 GO terms (Table S15). These results suggest that these selective sweeps may contribute to fibre quality improvement during cotton domestication.

### Genomic constitution of Ejing1 and Zhong 7263

**Figure 3:**
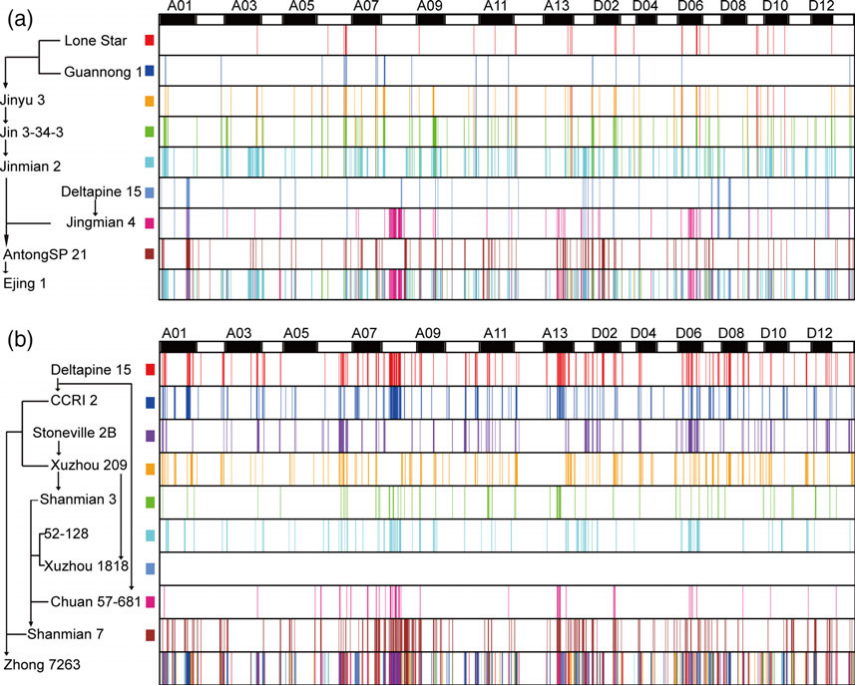
Genome flow of Ejing 1 and Zhong 7263. (a) Specific fragments collected in Ejing 1. (b) Specific fragments collected in Zhong 7263. Different colors represent different parents, and the corresponding color of each parent is noted on the vertical axis (left) together with the genetic pathway. The horizontal axis (top) indicates different chromosomes, A1–D13. Unique genetic segments in each parent are specifically passed according to the genetic pathway shown on the vertical axis.

Ejing 1, one of the parents of Ekangmian 9, is highly productive in the Yangtze River Basin and produces big bolls, large seeds and has a high lint percentage. Using identity by decent (IBD) detection and tracking, we found that 10.5% of the chromosomal sequences in Ejing 1 were derived from the parents (Fig. 3a and Table S16). These traceable fragments were differentially distributed in the A (59.60%) and D (40.40%) sub‐genomes (Figure S4a), indicating that a higher proportion of the A sub‐genome in Ejing 1 is derived from the parents. However, more genes in the D sub‐genome (2,845 genes), relative to the A sub‐genome (2651 genes), were found located in IBD segments (Figure S4b and Table S16). These genes were involved in 127 KEGG pathways (Table S17) and associated with 3,199 GO terms (Table S18). The distribution of genes located in IBD segments was highly correlated with the length of the segment (Figure S4b). These traceable fragments were found to overlap with 79 QTLs associated with key yield and quality trait loci, of which 42 and 37 QTLs were located in the A and D sub‐genomes, respectively (Table S19). In addition, Guannong 1 and Lone Star were used as progenitors of Ejing1 to investigate genetic inheritance of the IBD regions from generation to generation within the pedigree. The total length of the IBDs derived from Guannong 1 was respectively reduced by 18, 19 and 22 Mb from the generations Jinyu 3, Jin 3‐34‐3, Jinmian 2 to the progeny Ejing 1 (Table S20). The length of the IBD regions derived from Lone Star was reduced by 21 Mb on average after one generation (Table S20).

Zhong 7263, which is resistant to Fusarium wilt and tolerant to verticillium, was bred from the progeny of a combination of CCRI4 and Shan 5012 (Fig. 1a). IBD detection and tracking was used to identify and map 1828 traceable IBD fragments containing 8714 genes in Zhong 7263 (Fig. 3b and Table S21). These IBD fragments spanned 327.40 Mb, 60.5% of which was located in the A sub‐genome. Similarly, more IBD fragments were inherited with closer genetic relationships to Zhong 7263 in the pedigrees (Figure S4c). Most of the IBD fragments were found in the A sub‐genome; however, more genes were identified in the IBD segments mapped to the D sub‐genome, with 4,192 and 4,552 genes in the A and D sub‐genomes, respectively (Figure S4d and Table S21). These genes were predicted to be involved in 121 KEGG pathways (Table S22) and associated with 3538 GO terms (Table S23). These traceable fragments overlapped with 117 QTLs, of which 53 and 64 were located in the A and D sub‐genomes, respectively (Table S24). A large number of IBD fragments were retained among the various accessions (Table S25).

### Genomic constitution of Ekangmian 9

**Figure 4:**
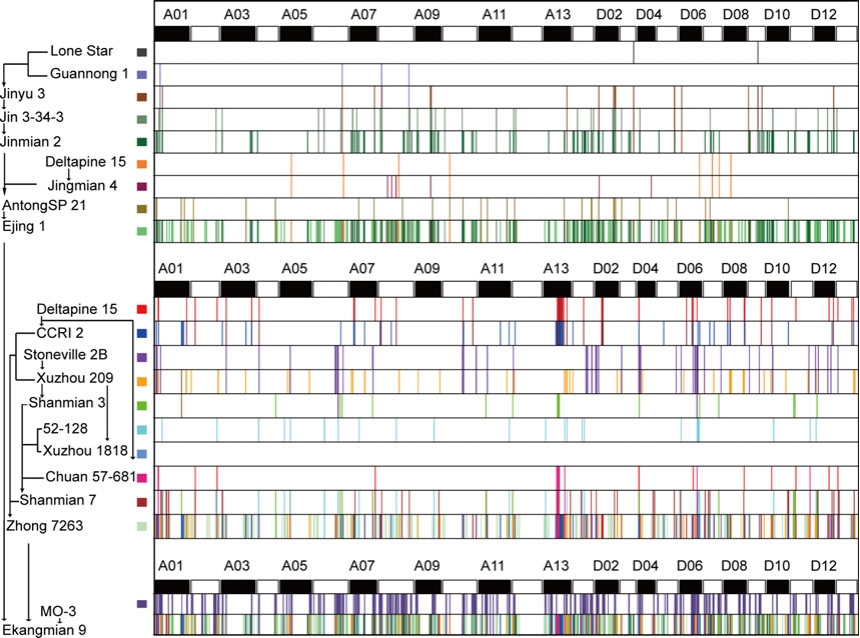
Genome flow of Ekangmian 9. Different colors represent different parents, and the corresponding color of each parent is noted on the vertical axis (left) together with the genetic pathway. The horizontal axis (top) indicates different chromosomes, A1–D13. Unique genetic segments in each parent are specifically passed to Ekangmian 9 according to the genetic pathway shown on the vertical axis.

Ekangmian 9, an important breeding variety, is a common parent of seven elite parents (Fig. 1a). Genetic component analysis showed that 29.23% of the genomic sequence of Ekangmian 9 could be traced back to parental origins (Fig. 4 and Table S26), including 11.79% from Zhong 7263, 11.44% from Ejing 1 and 6.00% from MO‐3 (Figure S5a), indicating that Zhong7263 and Ejing 1 were the major genetic contributors to Ekangmian 9. We further tracked the genetic origin of these fragments in the pedigree, and found that Ejing1 contributed 9.2% to the Ekangmian 9, which is the most contributor among all backbone parents, followed by the Zhong 7263 (8.21%) and MO‐3 (6.0%) (Fig. 4 and Table S27). Among the other backbone parents, Jinmian 2 (1.31%) is the most contributors of Ekangmian 9 (Figure S5b). A total of 17,616 genes were found in IBD fragments of Ekangmian 9, of which 10,262 and 7,354 genes were located in the D and A sub‐genomes, respectively (Table S26). These genes were predicted to be involved in 112 KEGG pathways (Table S28) and associated with 4033 GO terms (Table S29). These IBD fragments overlapped with 143 QTLs for yield or fibre quality traits, of which 90 and 53 QTLs were located in the D and A sub‐genomes, respectively (Table S30). Using a similar method, we further investigated the IBD fragments stably inherited from Ekangmian 9 to seven elite parents. The result showed that 10.2%~33.8% of the genomic sequences of these seven elite relatives were clearly inherited from Ekangmian 9 (Figure S5c). Similarly, we examined the IBD fragments of the seven elite parents stably inherited from Ejing 1, Zhong 7263 and MO‐3, and found that Ejing 1 and Zhong 7263 contributed more than MO‐3 (Figure S5d).

**Figure 5:**
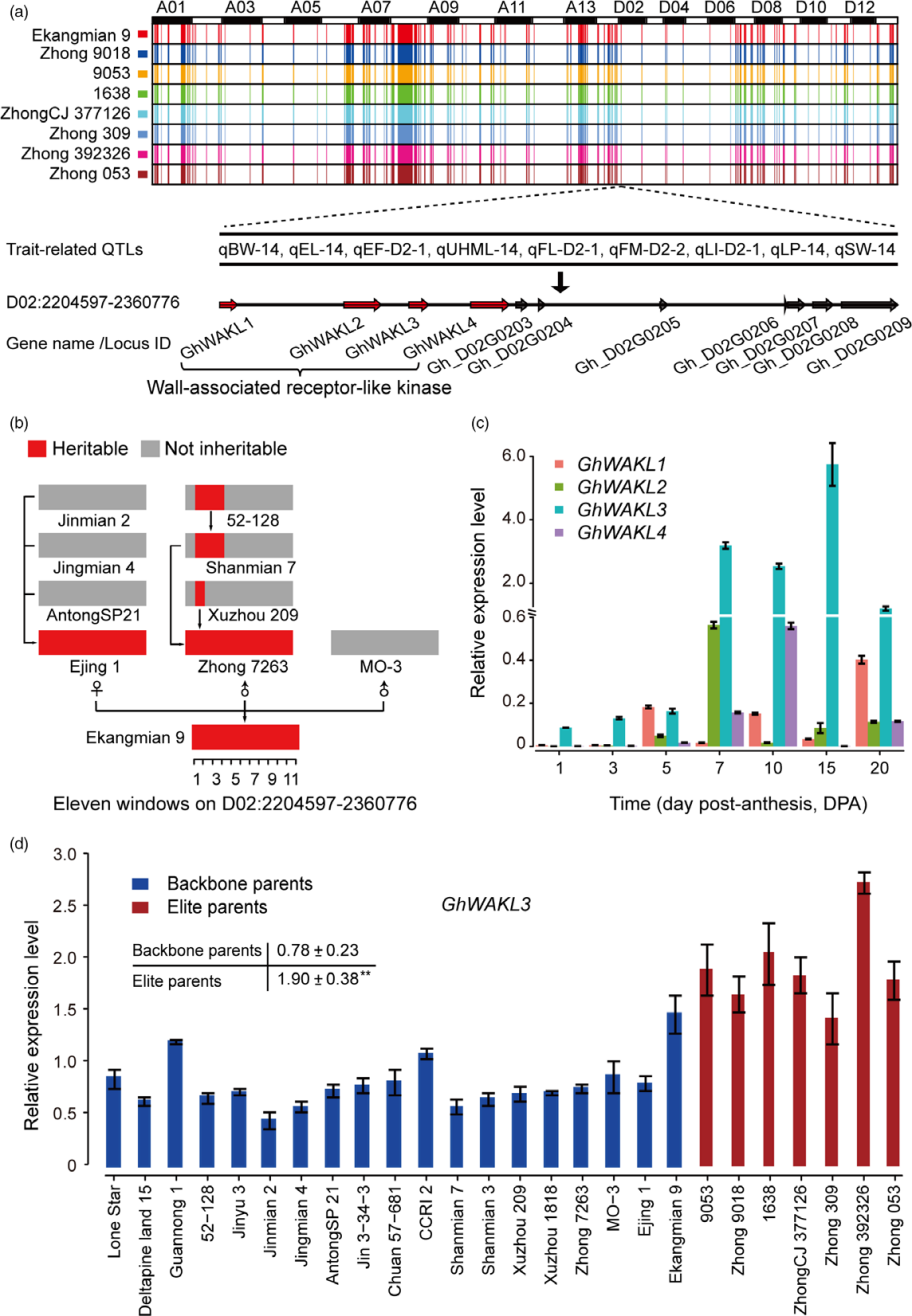
Identification of core genetic fragments and candidate genes related to fibre development. (a) Identification of core genetic fragments inherited from Ekangmian 9 in seven elite parents. Different colors corresponding to each parent are labelled on the vertical axis. D02:2204597‐2360776, a key candidate section containing nine QTLs. Arrows indicate genes located within this fragment. (b) Genetic tracking analysis of key candidate IBD segments (red). The segment was divided into 11 equal windows in length. (c) Quantitative RT‐PCR (qRT‐PCR) analysis of four cell wall related WAKL genes in TM‐1. Three biological and technical replicates were performed for qRT‐PCR experiments, and the error bars represent the mean values ± SE. Expression levels are relative to cotton *HIS3*. (d) Quantitative RT‐PCR (qRT‐PCR) analysis of *GhWAKL3* between backbone parents and elite parents (**P < 0.01, two‐sided *t*‐test).

Compared with their backbone parents, seven elite parents and Ekangmian 9 each displayed higher lint percentage (Fig. 1b), suggesting that some common genetic components were acquired during domestication and may play an important role in these lines. Therefore, we analysed the IBD fragments commonly possessed by the seven elite parental lines that were inherited from Ekangmian 9. As a result, a total of 526 common IBD fragments containing 1937 genes were identified, ranging in length from 12,522 bp to 1 836 814 bp, and a total length of 104 473 421 bp (Fig. 5a and Table S31). 1937 genes predicted to be involved in 103 KEGG pathways and associated with 2325 GO terms (Tables S32 and S33) were located in these common IBD fragments, and 1173 and 764 genes were located in the A and D sub‐genomes, respectively. The common IBD fragments that we identified were stably inherited from Ekangmian 9 and contained 26 GWAS sites and 28 QTLs associated with boll weight, number of bolls, lint percentage and fibre quality (Table S34). An important candidate fragment (D02: 2204597‐2360776) was found to possess up to 9 QTLs related to boll weight, lint percentage and seed index (Fig. 5a and Table S34). This segment contained a total of 11 genes, four of which were members of the cell wall‐associated kinase (WAK) and WAK‐like kinase (WAKL) family of receptor‐like kinase genes involved in plant cell wall development (Fig. 5a). Noticeably, cell wall development is intimately associated with fibre development (Wu *et al*., 2018), which is a key contributor to the increased lint percentage. When tracing this segment back to the parents of Ekangmian 9, we found that this genetic fragment can be found in both Ejing 1 and Zhong 7263, but not in the other parent, MO‐3 (Fig. 5b). Furthermore, this genetic fragment can also be partially traced back to Xuzhou 209, Shanmian 7 and 52‐128. However, it was not present in the parents of Ejing 1, indicating that this core genetic fragment may have been completely recreated in Ejing 1 during the breeding process.

### Expression profiles and candidate association of genes related to lint percentage

In order to further explore the function of the identified cell wall‐related genes, we analysed their expression levels during fibre development. The results suggested that these 4 WAKL genes were dominantly expressed during cotton fibre development, especially during secondary wall biosynthesis (Fig. 5c). Compared with the other three genes, *GhWAKL3* was highly up‐regulated during secondary cell wall biosynthesis, indicating that *GhWAKL3* may play an important role during cotton fibre development. These results were further verified by showing that expression levels of *GhWAKL3* were significantly higher in elite parents relative to backbone parents (Fig. 5d). Taken together, our data suggest that *GhWAKL3* plays a key role during cotton fibre development in our pedigree.

**Figure 6:**
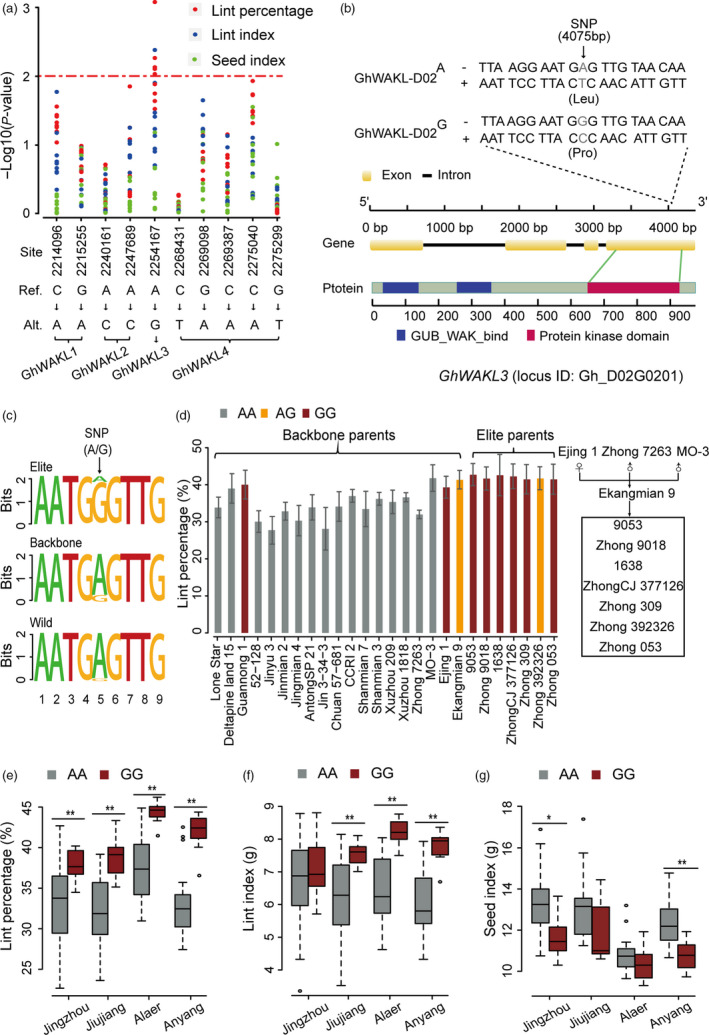
The GG genotype of *GhWAKL3* is a dominant mutation related to fibre development. (a) Correlation analysis between yield traits and non‐synonymous mutations present in candidate *GhWAKL* genes. Red dots, blue dots and green dots represent lint percentage, lint index and seed index, respectively. The x axis indicates the corresponding variation type, and the y axis represents the significance expressed as −log_10_ *P*. (b) Gene structure of *GhWAKL3* and the allelic variation identified within the pedigree. One non‐synonymous SNPs,resulting in an amino acid change from leucine to proline in the protein kinase domain of *GhWAKL3*, were shown to be associated with the GWAS signals for both higher lint yield and lint index. (c) Sequence logos of the non‐synonymous SNP site in the protein kinase domain of *GhWAKL3*. (d) Analysis of lint percentage of different genotypes of *GhWAKL3*. The x axis shows the different accessions. The genetic relationship between elite parents and their core parents (Ekangmian 9, Ejing 1, Zhong 7263, MO‐3) is displayed on the right. (e) Lint percentage (%), (f) Lint index (grams) and (g) seed index (grams) analyses of accessions with AA and GG genotypes. Centre line, median; box limits, upper and lower quartiles; whiskers, 1.5× the interquartile range (*P < 0.05, **P < 0.01, two‐sided *t*‐test).

To further study, the association between WAKL genes and lint percentage, ten non‐synonymous SNPs present in these genes, together with genotyping data from 258 diverse accessions, were used for additional analyses (Fang *et al*., 2017b). Our results showed that a SNP site located in *GhWAKL3* is the most significant site associated with lint percentage (Fig. 6a), suggesting that this alteration may be associated with this trait. This non‐synonymous SNP (SNP_D02_2254167) results in a nucleotide change from A to G, causing an amino acid substitution from leucine to proline in the protein kinase domain of *GhWAKL3* (Fig. 6b). In addition, this alteration leads to sequence variations that depart from the canonical motif (Fig. 6c), potentially disrupting or modifying the biochemical functions of *GhWAKL3*. 166 accessions with homozygous AA genotypes and 12 accessions with GG genotypes in SNP_D02_2254167 were used to further investigate the correlation between genotype and lint percentage. The results showed that SNP_D02_2254167 in the GG genotype is highly correlated with lint percentage (Figure S6a). Higher lint percentage and lint index were associated with these GG genotype accessions (Figure S6a,b). However, seed index was reduced in these lines (Figure S6c). In our pedigrees, all backbone parents, except for Guangnong1, Ejing1 and Ekangmian 9, were the AA genotype, whereas all elite parents were the GG genotype, except for Zhong 392326, which was the heterozygous genotype (AG genotype) (Fig. 6d). The genotype was closely correlated with yield, which was verified by the observation that GG genotype accessions exhibited a significantly higher lint percentage, lint index and lower seed index than those with the AA genotype across all locations (Fig. 6e,f,g). Taken together, these results suggest that a functional allele of *GhWAKL3* has been selected to improve lint percentage during modern breeding.

## Discussion

### Backbone parents play an important role in breeding

Genetically variant germplasm resources form the foundation of breeding‐based crop improvement (Shinada *et al*., 2014; Yamamoto *et al*., 2010). These differences have often spawned many genetic groups with their own representative varieties, providing excellent genetic backgrounds with special genetic characteristics, called backbone parents (Shinada *et al*., 2014). The utilization of backbone parents can effectively improve breeding efficiency during the breeding process, exemplified by the application of B73, Mo17, Chang7‐2, Zheng58 and Huangzaosi in maize breeding (Smith, 2007; Smith *et al*., 2004; Wu *et al*., 2014), and the use of Kitaake, Kyowa and Huanghuazhan in rice breeding (Shinada *et al*., 2014; Zhou *et al*., 2016a). Upland cotton was first introduced into China in the 19th century, and these original varieties comprised the original germplasm used for modern cotton breeding in China, especially germplasm originating from the United States (Fang *et al*., 2017b; Liu *et al*., 2003). Ekangmian 9, the core germplasm source used to produce our pedigrees, was the common parent of seven elite parents. These parental lines were then further used to breed eight excellent hybrid cotton cultivars (Fig. 1a), indicating that superb traits have been pooled from Ekangmian 9 during the improvement process of this pedigree.

### Pedigree‐based genome resequencing reveals artificial selection

Thus far, at least 47 draft plant genomes, including almost all of the important crop species, have been published (Hodzic *et al*., 2017). Using these genome sequences as precise references, it is now relatively straightforward to detect SNPs in many crop species (Rafalski, 2002). Next‐generation sequencing (NGS) technology has greatly reduced the labour and costs associated with resequencing studies (Sharma *et al*., 2017). The dN/dS ratio, phylogenetic branch points, zero diversity segments, genetic diversity and linkage disequilibrium were often used as indicators of artificial selection during evolution (Lai *et al*., 2010; Lam *et al*., 2010; Zhou *et al*., 2016a). In our study, the dN/dS ratio in the pedigree was higher than that observed in other crop plants, including sorghum, rice and soybean (Lam *et al*., 2010; Mace *et al*., 2013; McNally *et al*., 2009; Zheng *et al*., 2017), implying that our pedigrees have undergone very strong artificial selection during domestication, which is highly consistent with the phylogenetic divergence observed between the pedigree and wild cotton groups (Fig. 2b). The linkage disequilibrium levels of the pedigree were also notably increased in contrast to other cultivar groups in China (Fig. 2c). These results suggest that genomic diversity within the pedigree decreased under the stress of artificial selection, resulting in the formation of many haplotypes during pedigree improvement.

Although low diversity segments and selective sweeps revealed relative conservation of the genome in the pedigree, they represented different directions for breeding‐based improvement (Lai *et al*., 2010). Low diversity segments were often located in low recombination regions, which are unlikely to cause internal phenotypic differences within the pedigree (Gore *et al*., 2009). Selective sweep analysis has been widely used to identify putative domestication or improvement events in crops (Chen *et al*., 2017; Wang *et al*., 2017; Zhou *et al*., 2016a). Further, selective sweeps have been used to identify different selection signatures to reveal population‐specific selective directions in different kinds of populations (Chen *et al*., 2017). 647 zero diversity segments and 511 selective sweeps were identified in our pedigree (Tables S9 and S12). These selective sweeps overlapped with 79 QTLs associated with boll weight, boll number, lint percentage and fibre quality (Table S15). Among these QTLs, 31 and 48 are located in the A and D sub‐genomes, respectively, consistent with previous results demonstrating that the D sub‐genome might play a much more important role than the A sub‐genome in domestication (Wang *et al*., 2017).

### Traceable IBD segments revealed derivation of core segments and key trait loci during cotton improvement

In recent years, more and more studies have been performed to trace derivation of core segments and to exploit key trait loci during the breeding process by pedigree‐based genome resequencing (Chen *et al*., 2017; Lai *et al*., 2010; Wu *et al*., 2016; Zheng *et al*., 2017; Zhou *et al*., 2016a), due to their well‐defined genetic paths. In rice, the genome structure of Huanghuazhan was resolved by detecting traceable blocks throughout the whole genome using 50 kb bins and 85% identity as the threshold (Zhou *et al*., 2016a). In maize, IBD detection was performed to analyse the genetic diversities among foundation parents (Wu *et al*., 2016). In cotton, IBD analysis has shown that the genetic constitution of Deltapine 15 is more similar to modern cotton accession (Fang *et al*., 2017b). In our pedigree, Zhong 7263 contributed 9.2% to the Ekangmian 9 genome, followed by Ejing 1 (8.21%) and MO‐3 (6.00%). We will focus on the dissection of elite genes contributing to Ekangmian 9 from the three parents, and further elucidate the elite genes composition in Ekangmian 9 in the future. In our study, for the first time, we revealed selection by derivation of core segments and key trait loci during modern cotton improvement based on a complete pedigree. However, analysis of the exact function of IBD fragments in cotton remains challenging, but QTLs related with important traits may provide some support for elucidating the function of these fragments (Fang *et al*., 2017b). In our pedigree, a large number of QTLs were located in these traceable IBD segments, indicating that these IBD segments were closely related with traits (Tables S19, S24, S30 and S34).

Lint percentage is the major improved trait in our pedigree (Fig. 1b). We identified a key functional fragment contributing to lint percentage based on a combination of IBD and QTL analyses. Subsequently, the *GhWAKL3* gene, (homologous to AT1G69730 in Arabidopsis) located in one of these fragments, was found to be responsible for fibre development and is highly expressed during cotton fibre elongation. A non‐synonymous SNP in *GhWAKL3,* which results in an amino acid substitution from leucine to proline in the protein kinase domain of *GhWAKL3,* is significantly correlated with increased lint percentage (Fig. 6a), indicating that this amino acid may be important for the catalytic function of *GhWAKL3*. Lint percentage is a complex, quantitative trait regulated by multiple genes (Wang *et al*., 2017). Increased expression level of *GhWAKL3* and a non‐synonymous SNP resulted in amino acid change in *GhWAKL3* may play a dominant role contributing to the increase of lint percentage in cotton. Similar findings were found in previous reports that two non‐synonymous SNPs in *AIL6* gene are associated with higher lint yield and two SNP sites in Dof‐binding motif potential affect the transcript activity of *4CL* gene (Fang *et al*., 2017b; Wang *et al*., 2017). These core genome segments and the SNPs identified in the cell wall‐associated WAKL family identified herein can be used for future pedigree and molecular breeding efforts in cotton.

## Experimental procedures

### Sample preparation and DNA extraction

Twenty‐six accessions, including seven elite parents and nineteen backbone parents, were selected for Illumina sequencing (Fig. 1a). Seeds of the following varieties were provided by our laboratory and cultivated: Zhong 309, Zhong 053, Zhong 392326, 9053, Zhong 9018, 1638, ZhongCJ 377126 and Ekangmian 9 (parent). Seeds from the varieties used in this study are also available from the National Medium‐Term Gene Bank of Cotton (China). All samples were planted during the 2016 growing season in Jingzhou, Jiujiang, Alaer and Anyang in China to measure main agronomic and fibre quality traits. For DNA extraction, all seeds were grown in a climate controlled growth chamber under the following conditions: temperature, 37°C; humidity, 75%; and a 12 h day/12 h and night light regime. Fully expanded cotyledons were used for extraction of genomic DNA by a modified cetyl trimethylammonium bromide method (Paterson *et al*., 1993). Agarose gel electrophoresis was performed to verify the quality and purity of all DNA preparations. 1.5 μg of DNA was used for DNA library construction.

### Library construction and genome sequencing

A paired‐end sequencing library with an insert size of 350 bp was constructed for each cultivar. First, 350 bp DNA fragments were randomly generated for each variety using a hydrodynamic shearing platform (Covaris, https://covaris.com/ and ApplicationSupport@covaris.com). Next, the DNA fragments were treated according to the following manufacturer's specifications (Illumina): fragments were end repaired, polyA‐tails were added and they were ligated to paired‐end adaptors and amplified by PCR. Re‐sequencing was performed using the Illumina HiSeq 4000 platform in the Novogene Bioinformatics Institute (Beijing, China). In order to improve the reliability of the clean data, the raw sequence reads were trimmed using the following filtering procedure: removing reads with ≥10% unidentified nucleotides (N) and >10 nt aligned to the adapter; removing reads with >50% bases having phred quality <5; and removing putative PCR duplicates amplification in the library.

### Read alignments and SNP detection

Clean paired‐end reads were mapped to the reference genome of *G. hirsutum* L. acc. TM‐1 v1.1 using the BWA (Burrows‐Wheeler Aligner, version: v0.7.16) software platform and the following parameters: ‘mem ‐t 10 ‐k 32’ (Li and Durbin, 2009; Zhang *et al*., 2015). The **sequence alignment files (SAM) containing the overall mapping information created during the mapping process were counted, indexed and converted into binary BAM files using SAMtools software (version: 1.6, settings: ‐bS –t) (Li *et al*., 2009). Filtering was performed to improve the mapping results as follows: only the pair with the highest mapping score was retained when multiple read pairs had identical external coordinates, potential PCR (polymerase chain reaction) duplications were removed to reduce mismatch generated by PCR amplification before sequencing, and the results were trimmed to adhere to a minimum read depth (≥8 and ≤1000) and RMS mapping quality (≥20). The filtered BAM files were used to perform SNP calling for each sample. SNP detection was carried out using SAMtools software (Li *et al*., 2009). The following parameter was used for SNP calling: mpileup ‐m 2 ‐F 0.002 ‐d 1000. Genotype calling was also performed using SAMtools software with the minor allele frequency (MAF) set to greater than 0.05 and missing less than 0.2. All SNP annotations were obtained from general feature format files of the TM‐1 reference genome (Li *et al*., 2015).

### Population genetics analysis

To validate our population genetics analyses, previous SNP detection using 31 wild cotton cultivars was used in this study (Wang *et al*., 2017). SNPs of 31 wild cotton and 27 pedigree accessions were filtered with MAF = 0.05 and missing = 0.2. These filtered SNPs were used to generate a neighbour‐joining tree using PHYLIP (version:3.69) (Felsenstein, 1989). Linkage disequilibrium (LD) calculations were carried out using plink software with the following command: –ld‐window‐r2 0 –ld‐window 99999 –ld‐window‐kb 1000 (Purcell *et al*., 2007). This result was further used to calculate LD decay, and LD decay plots were generated using R scripts. Values of Nucleotide diversity (π) and population fixation statistics (Fst) were measured by calculating values using VCFtools (100 kb windows sliding 20 kb with the parameter: –window‐pi 100000 –window‐pi‐step 20000) (version: v0.1.14, http://vcftools.sourceforge.net) (Danecek *et al*., 2011). Domestication sweep windows between wild and pedigree varieties were detected using a 5% cutoff log10 (πwilds/πpedigrees). Cross population extended haplotype homozogysity (XP‐EHH) was used to identify the most affected selective sweeps by performing a genome‐wide analysis using 100 kb windows (Sabeti *et al*., 2007). Using the top 5% of XP‐EHH values as thresholds, the windows from the top 5% cut‐offs of log10 (π_wilds_/π_pedigrees_) values were further confirmed.

### IBD analysis

Strategies using sliding windows and calculation of SNP ratios were used for identification of IBD regions (Fang *et al*., 2017b; Jiao *et al*., 2012). A window size of 200 SNPs, with a step size of 20 SNPs, was used to perform genomic scans (Fang *et al*., 2017b). Lone Star, Deltapine 15, Stoneville 2B, Guannong 1 and 52‐128 were considered as exotic and/or older pedigree species. Genetic distances were calculated among these older species to filter out low‐diversity windows in the pedigree (Fang *et al*., 2017b). To detect IBD fragments in the pedigree, we calculated the SNP ratio between later generations and each parent. A window with the same SNP ratio ≥99% was considered as an inheritable IBD fragment in the pedigree.

### Association analysis of candidate genes with lint traits and gene expression analysis of candidate genes

GWAS data comprised of genotyping, lint percentage and seed index traits of 258 diverse accessions was released previously (Fang *et al*., 2017b). Non‐synonymous mutant SNP sites located in candidate genes were identified and extracted. These SNP sites existed in both the GWAS population and our pedigrees. We performed association analysis between these non‐synonymous SNPs and lint traits based on kinship coefficients and the population structure of the GWAS population (principal component analysis (PCA) + K model). The kinship coefficients (K) and PCA were estimated using TASSEL 5.0 software (Bradbury *et al*., 2007). In addition, candidate gene expression levels in TM‐1 were investigated ‐1, 0, 1, 3, 5, 7, 10, 15 and 20 days post‐anthesis (DPA) by real‐time quantitative polymerase chain reaction (qRT‐PCR).

### Statistical analysis

To measure phenotypic differences between backbone and elite parents, EXCEL 2013 was used to perform *F*‐tests and two‐tailed Student's *t*‐tests with a significant level of 0.05 and an extremely significant level of 0.01.

## Author contributions

Daigang Yang designed the project. Daigang Yang and Xiongfeng Ma coordinated the project. Zhenyu Wang, Wei Li, Xiaojian Zhou, Yangai Liu, Zhongying Ren, Kehai Zhou, Wensheng Zhang and Xiaoyu Pei performed the field experiments and investigated phenotypic data. Zhenyu Wang, Wei Li, Kunlun He, Fei Zhang, Junfang Liu and Wenyu Ma statistically analysed the phenotypic data. Zhenyu Wang carried out DNA extraction, next‐generation sequencing data analysis, IBD detection and figure production. Zhenyu Wang and Wei Li performed selective evolution analysis and association analysis of candidate genes. Guanghui Xiao analysed of real‐time quantitative polymerase chain reaction (qRT‐PCR) data and drew its figure. Zhenyu Wang and Wei Li wrote the manuscript, which was revised by Guanghui Xiao, Yuzhou Zhang and Daigang Yang.

## Conflict of Interest

The authors declare no competing financial interests.

## Supporting information


**Figure S1** Analysis of lint percentage, lint index and seed index in 26 accessions cultivated in four locations in China. (a) Lint percentage, (b) lint index and (c) seed index analyses across 26 accessions grown in Jingzhou, Jiujiang, Alaer and Anyang.
**Figure S2** The distribution of single‐nucleotide polymorphisms (SNPs) in the pedigree. (a) Analysis of SNP number, (b) and identification of synonymous and non‐synonymous SNPs and (c) stop gain and stop loss SNPs present in the pedigree.
**Figure S3** Comparative analysis of selective improvement sweeps in wild type and pedigree cotton accessions along the genome. A genome‐wide threshold of 1.38 was defined by the top 5% of the log_10_(π_wilds_/π_pedigrees_).
**Figure S4** Distribution of IBD fragments in the pedigree. (a) Length of IBD fragments in Ejing 1 inherited from multiple parents. (b) Number of genes located in IBD fragments of Ejing 1. (c) Length of IBD fragments in Zhong 7263 inherited from multiple parents. (d) Number of genes located in IBD fragments of Zhong 7263. The x axis indicates the length of IBD fragments (a,c) or the number of genes (b,d) affected in the pedigree. The y axis indicates the accessions used in this study.
**Figure S5** Analysis of Ekangmian 9 and identification of IBD regions in seven elite parents. (a) The genomic proportion of Ekangmian 9 inherited from Ejing 1, Zhong 7263 and MO‐3. The blue, green, purple and red bars represent the genetic makeup of Zhong 7263, MO‐3, Ejing 1 and unknown genetic components, respectively. (b) The genetic contribution of backbone parents to Ekangmian 9. (c) The genetic contribution of Ekangmian 9 to 7 elite parents. The blue bar represents the genetic makeup of Ekangmian 9 and the red bar represents unknown genetic components. (d) The genetic constitution of seven elite parents from Ekangmian 9, Ejing 1, Zhong 7263 and MO‐3.
**Figure S6** Correlation between two different genotypes of *GhWAKL3* and yield traits across nine environments. (a) Lint percentage, (b) lint index and (c) seed index analyses of accessions with AA and GG genotypes. Centre line, median; box limits, upper and lower quartiles; whiskers, 1.5 × the interquartile range. All accessions were grown in three locations in China for 3 years. an, ku and nan represent Anyang, Kuche and Nanjing, respectively. *P < 0.05, **P < 0.01, two‐sided t‐test.

**Table S1** 106 phenotype sets measured in the pedigree accessions.
**Table S2** Phenotypic differences between elite parents and backbone parents.
**Table S3** Results of sequencing data and alignments.
**Table S4** Results of SNP annotation.
**Table S5** Large effect SNPs and affected genes.
**Table S6** KEGG pathway enrichment analyses for effected SNP genes.
**Table S7** Gene ontology term enrichment analyses for effected SNP genes.
**Table S8** Distribution of SNP density on genome.
**Table S9** Zero diversity segments and genes contained within them.
**Table S10** KEGG pathway enrichment analyses for genes contained in zero diversity regions.
**Table S11** Gene ontology term enrichment analyses for genes contained in zero diversity regions.
**Table S12** Statistical analysis of potential selective sweeps and genes contained within them.
**Table S13** Cotton QTLs overlapping with putative selective sweeps.
**Table S14** KEGG pathway enrichment analyses for genes contained in potential selective sweeps.
**Table S15** Gene ontology term enrichment analyses for genes contained in potential selective sweeps.
**Table S16** Genetic constitution of Ejing 1.
**Table S17** KEGG pathway enrichment analyses for genes overlapped with traceable IBD fragments of Ejing 1.
**Table S18** Gene ontology term enrichment analyses for genes overlapped with traceable IBD fragments of Ejing 1.
**Table S19** Cotton QTLs overlapping with IBD segments of Ejing 1.
**Table S20** The length of IBD segments passed between materials during Ejing 1 improvement.
**Table S21** Genetic constitution of Zhong 7263.
**Table S22** KEGG pathway enrichment analyses for genes overlapped with traceable IBD fragments of Zhong 7263.
**Table S23** Gene ontology term enrichment analyses for genes overlapped with traceable IBD fragments of Zhong 7263.
**Table S24** Cotton QTLs overlapping with IBD segments of Zhong 7263.
**Table S25** Length of IBD segments passed between materials during improvement of Zhong 7263.
**Table S26** Genetic constitution of Ekangmian 9.
**Table S27** The ratio of genome contribution of parents to Ekangmian 9.
**Table S28** KEGG pathway enrichment analyses for genes overlapped with traceable IBD fragments of Ekangmian 9.
**Table S29** Gene ontology term enrichment analyses for genes overlapped with traceable IBD fragments of Ekangmian 9.
**Table S30** Cotton QTLs overlapping with IBD segments of Ekangmian 9.
**Table S31** Common IBD fragments inherited from Ekangmian 9 to seven elite parents.
**Table S32** KEGG pathway enrichment analyses for genes overlapped with traceable common IBD fragments of seven elite parents.
**Table S33** Gene ontology term enrichment analyses for genes overlapped with traceable common IBD fragments of seven elite parents.
**Table S34** Cotton QTLs overlapping with common IBD fragments of seven elite parents.

## Data Availability

All the sequence data sets generated during the current study are available in the NCBI Sequence Read Archive (SRA) under accession SRP145619.
