# Supplementary material for: Resequencing core accessions of a pedigree identifies derivation of genomic segments and key agronomic trait loci during cotton improvement
Source: Plant Biotechnol J. 2018 Oct 18;17(4):762–75. doi: 10.1111/pbi.13013 (PMC6419577; doi:10.1111/pbi.13013)
Supplement: Supplementary file 1 — Figure S1 Analysis of lint percentage, lint index and seed index in 26 accessions cultivated in four locations in China. (a) Lint percentage, (b) lint index and (c) seed index analyses across 26 accessions grown in Jingzhou, Jiujiang, Alaer and Anyang. Figure S2 The distribution of single‐nucleotide polymorphisms (SNPs) in the pedigree. (a) Analysis of SNP number, (b) and identification of synonymous and non‐synonymous SNPs and (c) stop gain and stop loss SNPs present in the pedigree. Figure S3 Comparative analysis of selective improvement sweeps in wild type and pedigree cotton accessions along the genome. A genome‐wide threshold of 1.38 was defined by the top 5% of the log10(πwilds/πpedigrees). Figure S4 Distribution of IBD fragments in the pedigree. (a) Length of IBD fragments in Ejing 1 inherited from multiple parents. (b) Number of genes located in IBD fragments of Ejing 1. (c) Length of IBD fragments in Zhong 7263 inherited from multiple parents. (d) Number of genes located in IBD fragments of Zhong 7263. The x axis indicates the length of IBD fragments (a,c) or the number of genes (b,d) affected in the pedigree. The y axis indicates the accessions used in this study. Figure S5 Analysis of Ekangmian 9 and identification of IBD regions in seven elite parents. (a) The genomic proportion of Ekangmian 9 inherited from Ejing 1, Zhong 7263 and MO‐3. The blue, green, purple and red bars represent the genetic makeup of Zhong 7263, MO‐3, Ejing 1 and unknown genetic components, respectively. (b) The genetic contribution of backbone parents to Ekangmian 9. (c) The genetic contribution of Ekangmian 9 to 7 elite parents. The blue bar represents the genetic makeup of Ekangmian 9 and the red bar represents unknown genetic components. (d) The genetic constitution of seven elite parents from Ekangmian 9, Ejing 1, Zhong 7263 and MO‐3. Figure S6 Correlation between two different genotypes of GhWAKL3 and yield traits across nine environments. (a) Lint percentage, (b) lint index a [file PBI-17-762-s001.pdf]

Supplementary Figures: Figure S1 to S6

Resequencing core accessions of a pedigree identifies derivation of genomic segments and key agronomic trait loci during cotton improvement

Xiongfeng Ma[1, †], Zhenyu Wang[1, †], Wei Li[1, †], Yuzhou Zhang[2, †], Xiaojian Zhou[1], Yangai Liu[1], Zhongying Ren[1], Xiaoyu Pei[1], Kehai Zhou[1], Wensheng Zhang[1], Kunlun He[1], Fei Zhang[1], Junfang Liu[1], Wenyu Ma[1], Guanghui Xiao[2]*, Daigang Yang[1]*

[1] State Key Laboratory of Cotton Biology, Institute of Cotton Research, Chinese Academy of Agricultural Sciences, Anyang 455000, China.

[2] Key Laboratory of the Ministry of Education for Medicinal Plant Resources and Natural Pharmaceutical Chemistry, National Engineering Laboratory for Resource Development of Endangered Crude Drugs in the Northwest of China, College of Life Sciences, Shaanxi Normal University, Xi'an 710119, China.

* To whom correspondence should be addressed. Tel: +86 0372 2562215; Email: yangdaigang@caas.cn. Correspondence may also be addressed to Guanghui Xiao, Email: guanghuix@snnu.edu.cn

[†] The authors wish it to be known that, in their opinion, the first four authors should be regarded as joint First Authors.

Present Address: Daigang Yang, Institute of Cotton Research, Chinese Academy of Agricultural Sciences, Anyang 455000, China, and Guanghui Xiao, College of Life Sciences, Shaanxi Normal University, Xi'an 710119, China.

This file includes: Figure S1 to S6.

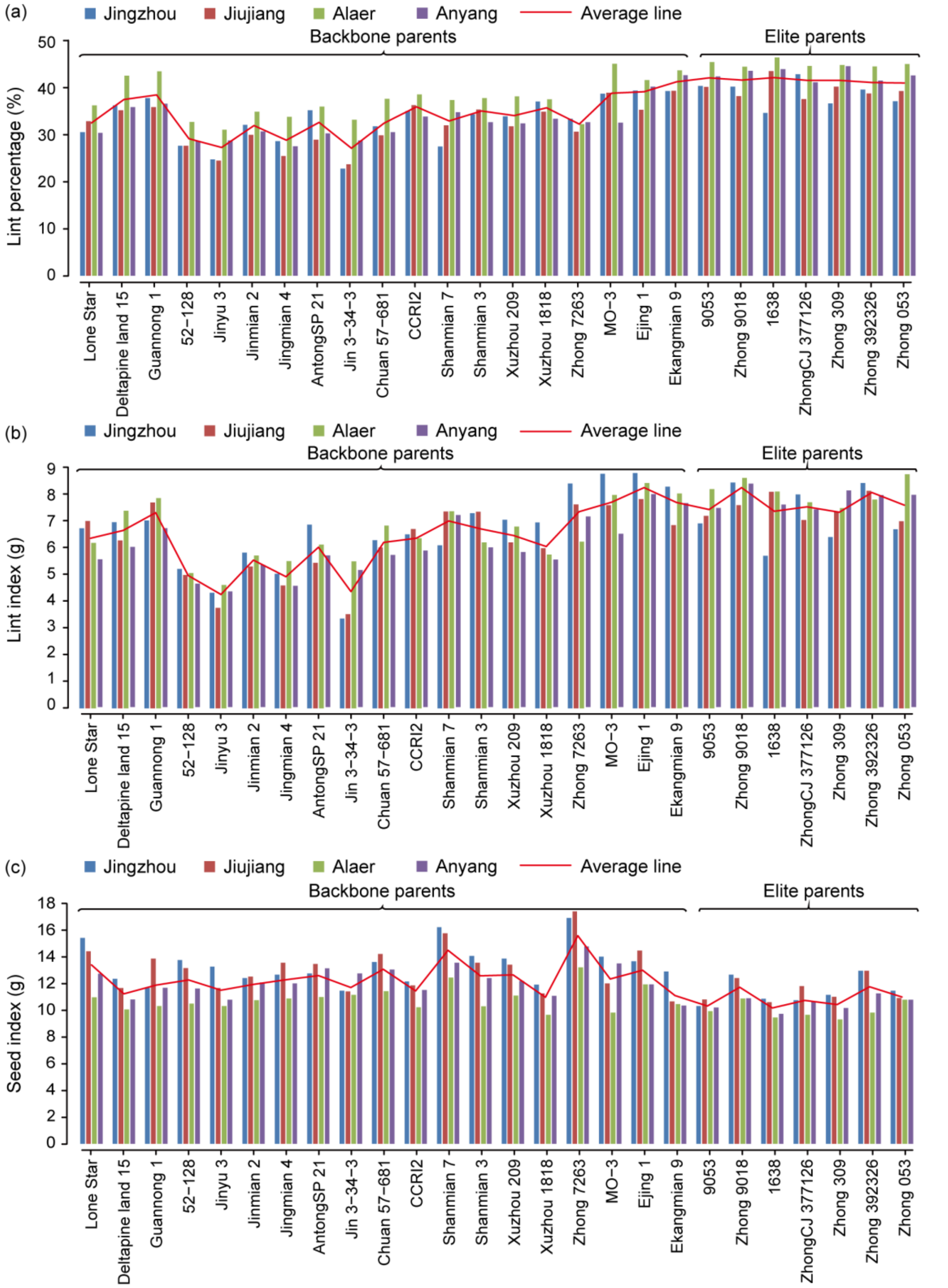

Figure S1. Analysis of lint percentage, lint index, and seed index in 26 accessions cultivated in 4 locations in China. (a) Lint percentage, (b) lint index, and (c) seed index analyses across 26 accessions grown in Jingzhou, Jiujiang, Alaer, and Anyang.

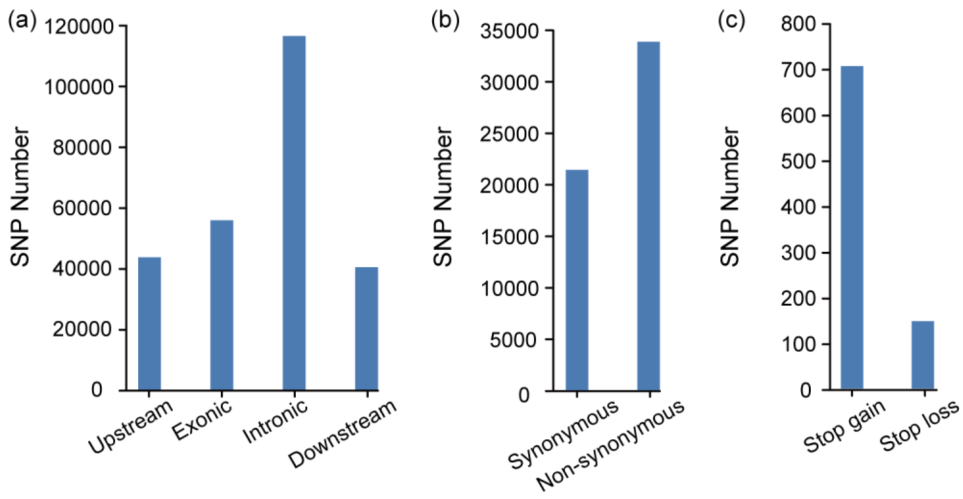

Figure S2. The distribution of single-nucleotide polymorphisms (SNPs) in the pedigree. (a) Analysis of SNP number, (b) and identification of synonymous and non-synonymous SNPs and (c) stop gain and stop loss SNPs present in the pedigree.

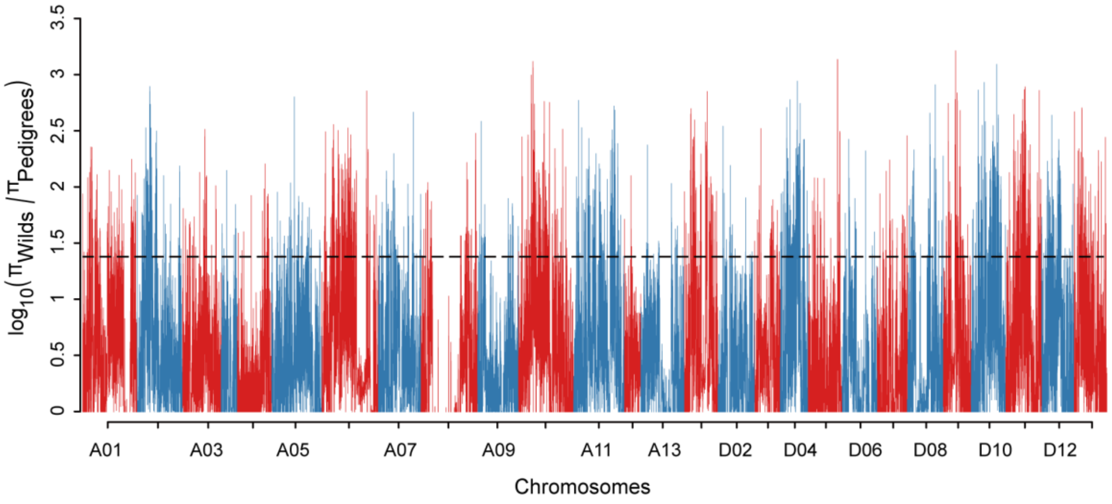

Figure S3. Comparative analysis of selective improvement sweeps in wild type and pedigree cotton acessions along the genome. A genome-wide threshold of 1.38 was defined by the top 5% of the $\log_{10}(\pi_{wilds} / \pi_{pedigrees})$.

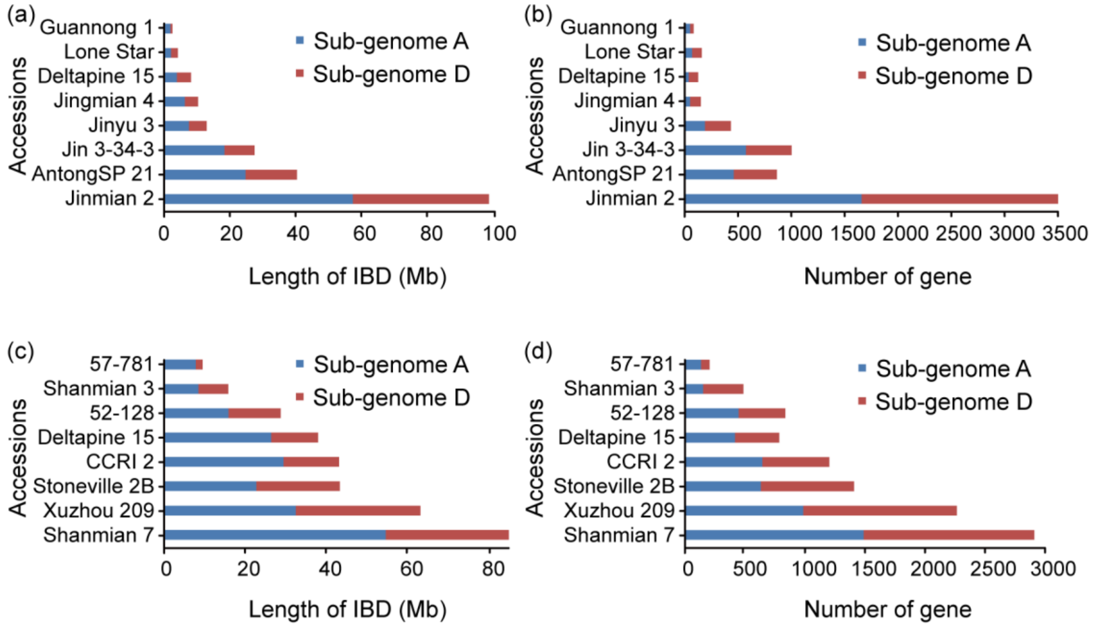

Figure S4. Distribution of IBD fragments in the pedigree. (a) Length of IBD fragments in Ejing 1 inherited from multiple parents. (b) Number of genes located in IBD fragments of Ejing 1. (c) Length of IBD fragments in Zhong 7263 inherited from multiple parents. (d) Number of genes located in IBD fragments of Zhong 7263. The x axis indicates the length of IBD fragments (a,c) or the number of genes (b,d) affected in the pedigree. The y axis indicates the accesssions used in this study.

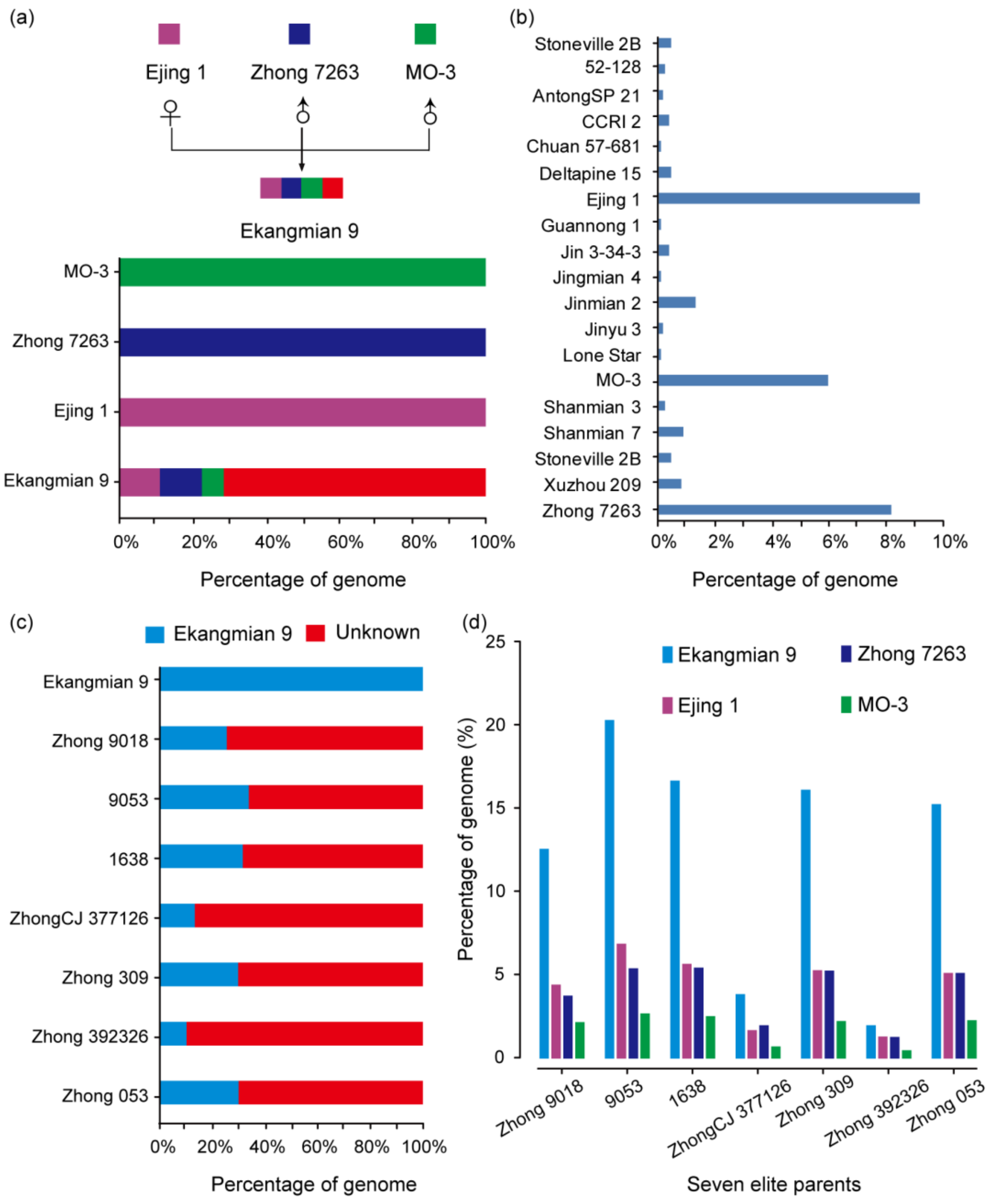

Figure S5. Analysis of Ekangmian 9 and identification of IBD regions in 7 elite parents. (a) The genomic proportion of Ekangmian 9 inherited from Ejing 1, Zhong 7263, and MO-3. The blue, green, purple, and red bars represent the genetic makeup of Zhong 7263, MO-3, Ejing 1, and unknown genetic components, respectively. (b) The genetic contribution of backbone parents to Ekangmian 9. (c) The genetic contribution of Ekangmian 9 to 7 elite parents. The blue bar represents the genetic makeup of Ekangmian9 and the red bar represents unknown genetic components. (d) The genetic constitution of 7 elite parents from Ekangmian 9, Ejing 1, Zhong 7263, and MO-3.

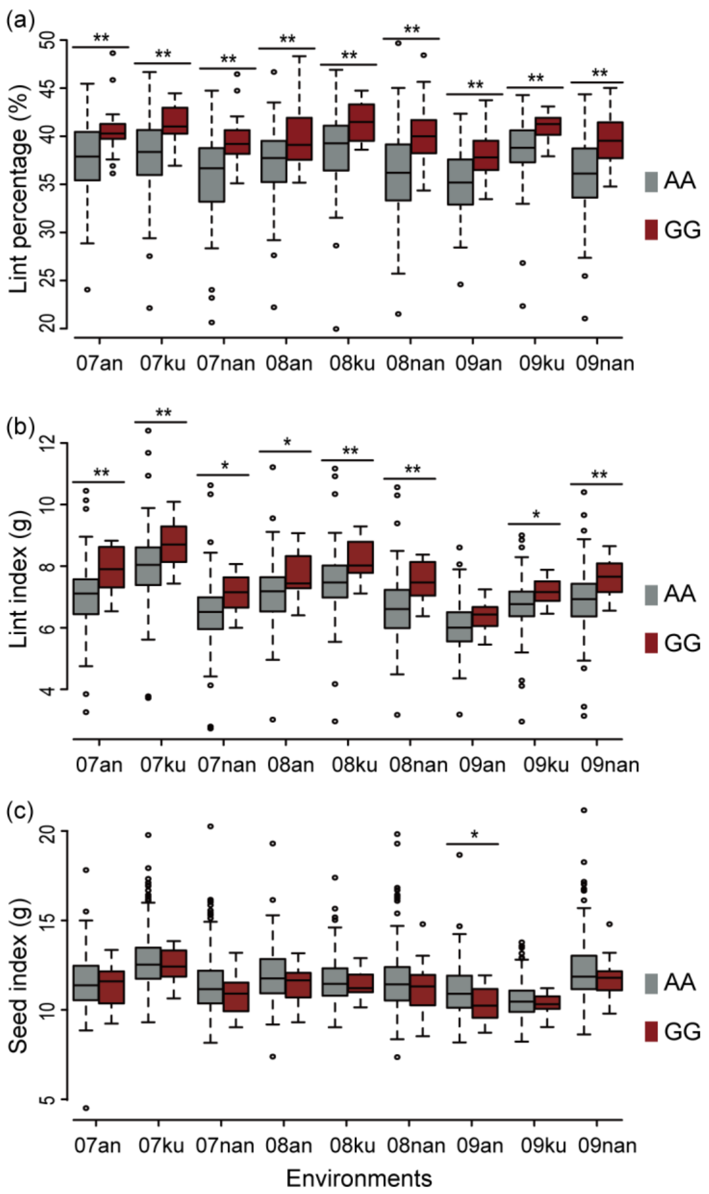

Figure S6. Correlation between two different genotypes of *GhWAKL3* and yield traits across nine environments. (a) Lint percentage, (b) lint index, and (c) seed index analyses of accessions with AA and GG genotypes. Center line, median; box limits, upper and lower quartiles; whiskers, 1.5× the interquartile range. All accessions were grown in three locations in China for three years. an, ku and nan represent Anyang, Kuche, and Nanjing, respectively. *P < 0.05, **P < 0.01, two-sided t-test.
